# Supplementary material for: Elicitor Specific Mechanisms of Defence Priming in Oak Seedlings Against Powdery Mildew
Source: Plant Cell Environ. 2025 Feb 25;48(6):4455–74. doi: 10.1111/pce.15419 (PMC12050401; doi:10.1111/pce.15419)
Supplement: Supplementary file 1 — Supporting information. [file PCE-48-4455-s013.pdf]

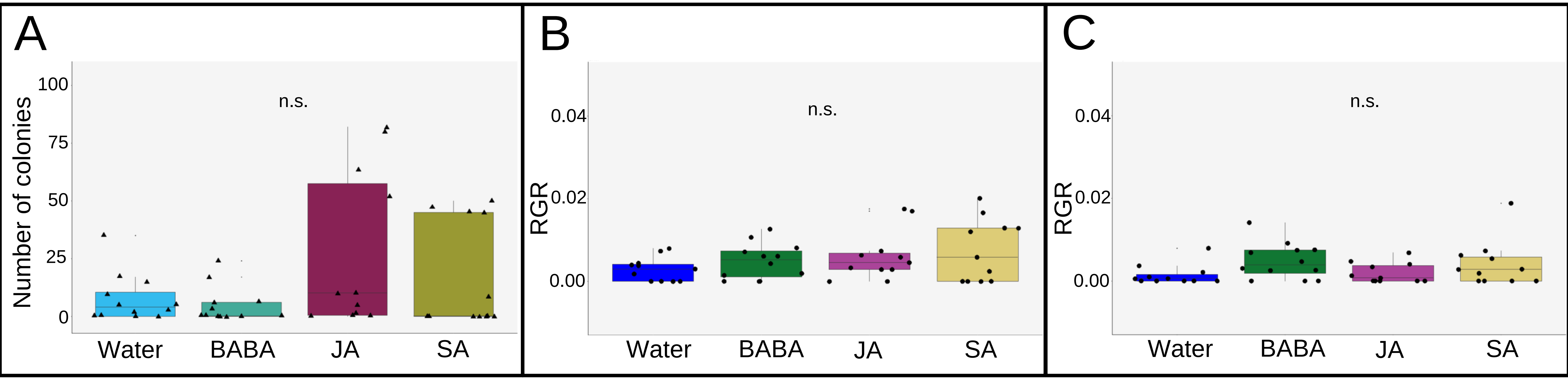

Figure S1: Growth, disease and resistance phenotypes. A) Number of colonies at 14 dpi. B) Relative growth rate (RGR) per day for diameter. C) Relative growth rate (RGR) per day for leaf length. For A,B and C Kruskal-Wallis was not significant ($p > 0.05$, $n = 11-12$).
