# Supplementary material for: Elicitor Specific Mechanisms of Defence Priming in Oak Seedlings Against Powdery Mildew
Source: Plant Cell Environ. 2025 Feb 25;48(6):4455–74. doi: 10.1111/pce.15419 (PMC12050401; doi:10.1111/pce.15419)
Supplement: Supplementary file 3 — Supporting information. [file PCE-48-4455-s002.pdf]

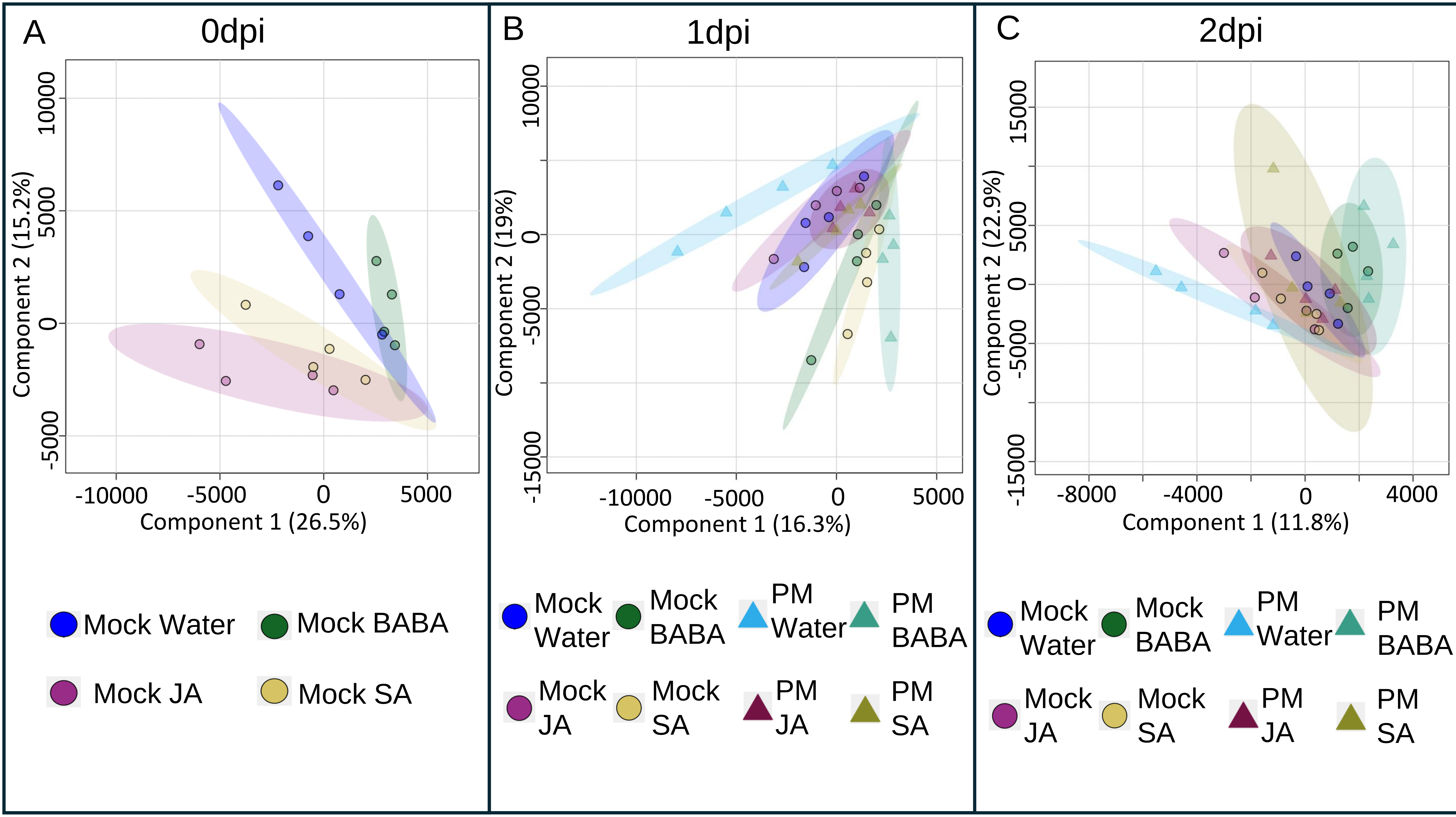

Figure S3: Metabolome PLS-DA plots. A) Partial Least-Squares Discriminant Analysis (PLS-DA) scores plot for mock plants at 0 dpi. B-C) PLS-DA scores plots for mock and infected plants at 1 dpi and 2dpi, respectively.
