# Supplementary material for: Elicitor Specific Mechanisms of Defence Priming in Oak Seedlings Against Powdery Mildew
Source: Plant Cell Environ. 2025 Feb 25;48(6):4455–74. doi: 10.1111/pce.15419 (PMC12050401; doi:10.1111/pce.15419)
Supplement: Supplementary file 4 — Supporting information. [file PCE-48-4455-s010.pdf]

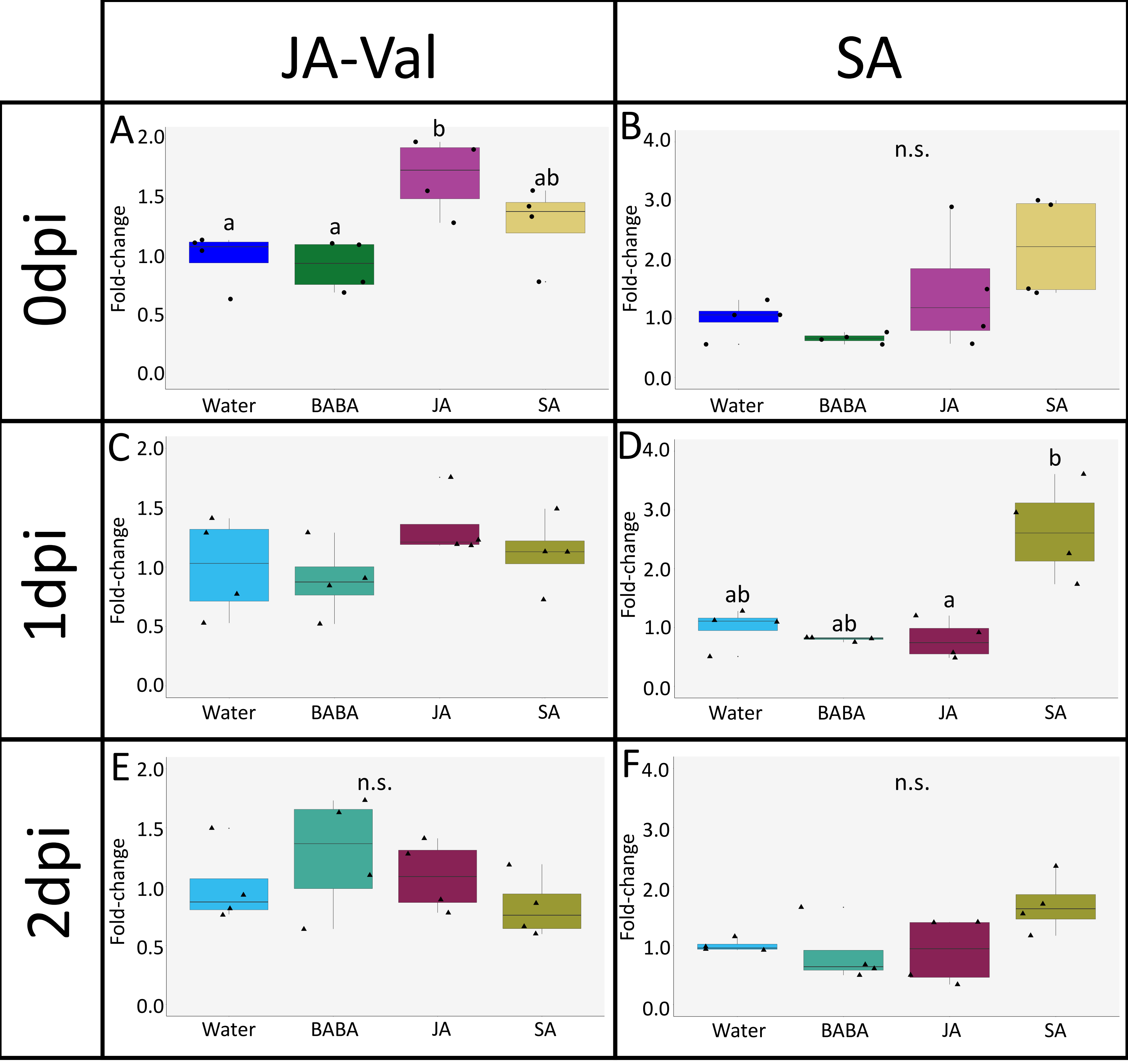

Figure S4: Fold-changes for endogenous hormones following elicitor treatment. A) JA-Val fold-changes compared to water mock at 0 dpi. Letters represent statistically significant differences (ANOVA + Tukey post hoc test, $p \leq 0.05$, n = 4). B) SA fold-changes compared to water mock at 0 dpi. Welch ANOVA was not significant ($p > 0.05$, n = 4). C) JA-Val fold-changes compared to water PM at 1dpi. ANOVA was not significant ($p > 0.05$, n = 4). D) SA fold-changes compared to water PM at 1dpi. Letters represent statistically significant differences (Welch ANOVA + Dunnet's T3, $p \leq 0.05$, n = 4). E) JA-Val fold-changes compared to water PM at 2dpi. ANOVA was not significant ($p > 0.05$, n = 4). F) SA fold-changes compared to water PM at 1dpi. ANOVA was not significant ($p > 0.05$, n = 4). JA-Val = jasmonic acid-valine. SA = salicylic acid.
